# Dual-Color Fluorescence Imaging of Magnetic Nanoparticles in Live Cancer Cells Using Conjugated Polymer Probes

**DOI:** 10.1038/srep22368

**Published:** 2016-03-02

**Authors:** Minjie Sun, Bin Sun, Yun Liu, Qun-Dong Shen, Shaojun Jiang

**Affiliations:** 1 Department of Pharmaceutics, China Pharmaceutical University, Nanjing, 210009, China; 2 Department of Polymer Science & Engineering and Key Laboratory of High Performance Polymer Materials & Technology of MOE, Collaborative Innovation Center of Chemistry for Life Sciences, School of Chemistry & Chemical Engineering, Nanjing University, Nanjing 210023, China; 3 Department of Pathology and Laboratory of Electron Microscopy, Jinling Hospital, Nanjing 210002, China

## Abstract

Rapid growth in biological applications of nanomaterials brings about pressing needs for exploring nanomaterial-cell interactions. Cationic blue-emissive and anionic green-emissive conjugated polymers are applied as dual-color fluorescence probes to the surface of negatively charged magnetic nanoparticles through sequentially electrostatic adsorption. These conjugated polymers have large extinction coefficients and high fluorescence quantum yield (82% for PFN and 62% for ThPFS). Thereby, one can visualize trace amount (2.7 μg/mL) of fluorescence-labeled nanoparticles within cancer cells by confocal laser scanning microscopy. Fluorescence labeling by the conjugated polymers is also validated for quantitative determination of the internalized nanoparticles in each individual cell by flow cytometry analysis. Extensive overlap of blue and green fluorescence signals in the cytoplasm indicates that both conjugated polymer probes tightly bind to the surface of the nanoparticles during cellular internalization. The highly charged and fluorescence-labeled nanoparticles non-specifically bind to the cell membranes, followed by cellular uptake through endocytosis. The nanoparticles form aggregates inside endosomes, which yields a punctuated staining pattern. Cellular internalization of the nanoparticles is dependent on the dosage and time. Uptake efficiency can be enhanced three-fold by application of an external magnetic field. The nanoparticles are low cytotoxicity and suitable for simultaneously noninvasive fluorescence and magnetic resonance imaging application.

Rapid development of nanotechnologies has brought about tremendous nanoscale materials with distinct properties. Successful exploration of biological and medical applications of nanomaterials includes biosensing, cellular imaging, bio-separation, clinical diagnosis, medical therapy, and drug delivery^1^,^2^,^3^,^4^,^5^. In spite of many exciting results, great efforts are desired for improving the fundamental understanding of cell-nanomaterial interactions or evaluating potential hazard of the nanomaterials^6^,^7^,^8^,^9^,^10^,^11^. Researches on cellular responses to nanomaterials are at a rather primitive level and receive insufficient attentions. Hundreds of distinct cell types and thousands of cell lines in the adult human body could be the targets of the nanomaterials. For tracking nanomaterials in live cells, electron microscopy, surface plasmon resonance, and magnetic resonance imaging have proven to be extraordinarily useful, while these methods are time-consuming or not widely applicable to numerous types of nanomaterials with remarkable diversity.

Fluorescence techniques, including confocal optical microscopy and flow cytometry, are the most versatile modalities in biology and contribute important insights into cell-nanomaterials interactions with high sensitivity^12^,^13^,^14^,^15^. They take the advantages of noninvasive imaging of cells and tissues, the availability of plentiful fluorescence probes to label specific gene products or to visualize molecular interactions inside cells, high time (nanosecond) resolution to trace movement of the nanomaterials inside cells, and high spatial resolution to analyze individual cells. It is also capable of rapid (up to thousands of cells per second) single-cell fluorescence analysis by flow cytometry technique.

Conjugated polymers are of interest as very promising fluorescence probes. They are high-efficient light-harvesting and emitting molecules with molar extinction coefficients two to three orders of magnitude than those of organic dyes, and have fluorescence quantum yield as high as 50–90%. As a result, fluorescence measurement can be carried out in the nanomolar or picomolar concentration range of the conjugated polymers. Conjugated polymers show remarkable photophysical properties of highly efficient transportation of electronic excited state, high photo-stability, high emission rates, and little blinking as compared to organic dyes and semiconductor nanocrystals^16^. These properties make conjugated polymers powerful tools for fluorescence sensing and imaging applications. The emission signals of the conjugated polymers are highly sensitive to the presence of the analytes in nanomolar or subnanomolar quantities, and thereby suitable for application in ultra-trace sensors^17^,^18^. By introduction of ionic side-groups, conjugated polymers, also known as conjugated polyelectrolytes, achieve water-solubility^19^. The general sensory concept could thereby be extended to biological systems based on the fluorescence signal modulation of the conjugated polyelectrolytes in response to their electrostatic interaction with oppositely charged biomolecules^20^. It has driven interest in high-sensitive and rapid-response fluorescence sensors for polynucleotides (DNA or RNA), proteins or peptides (enzymes or antibodies), and so on^21^,^22^,^23^,^24^,^25^,^26^. Conjugated polymers have thus emerged as candidates for detecting gene mutations, monitoring gene transfer, high-throughput drug screening, and medical diagnostics^27^,^28^.

Most recently, researchers have generated tremendous interests in fluorescence microscopy imaging of biological cells by conjugated polymers^29^,^30^,^31^,^32^,^33^,^34^ or their nanoparticles prepared by emulsion, precipitation, polymerization techniques, or bacteria-mediated assemblies^35^,^36^,^37^,^38^,^39^,^40^,^41^. The intense fluorescence, lack of cytotoxicity, and efficient cellular uptake make the conjugated polymers highly advantageous as labels for fluorescence imaging and flow cytometry analysis of biological cells. Nanoscale 3D tracking, which is extraordinarily useful for investigating a wide variety of cellular processes, such as molecule transport and membrane dynamics, of the conjugated polymers has also been demonstrated^42^.

In the paper, we describe dual-color fluorescence labeling of magnetic nanoparticles (MP) through alternatively electrostatic adsorption of two conjugated polyelectrolytes with different optical band gaps, followed by tracking the interaction between fluorescence-labeled magnetic nanoparticles and human hepatoma carcinoma (liver cancer) cells (Fig. 1). The biomedical uses of magnetic nanoparticles have been well documented, including magnetic resonance imaging for tracking spatial location and migration of the cells within organs and tissues, separation of the cells, and hyperthermic treatment of cancer cells^43^. Magnetic/fluorescence dual-functional nanoparticles have also been developed for biomedical applications^44^. Our strategy for developing magnetic nanoparticles with dual-wavelength fluorescence emission is based on convenient and well-established electrostatic assembly technique^45^. As shown in Fig. 1, cationic blue-emissive conjugated polymer (PFN) and anionic green-emissive conjugated polymer (ThPFS) are sequentially attached on the surface of negatively charged magnetic nanoparticles, leading to the formation of ultrathin fluorescent film with three-layer structure. Conjugated polyelectrolytes are ideal surface coating, because they can satisfy the following requirements: (i) maintaining water solubility of the magnetic nanoparticles and preventing them from aggregation during the long-term storage; (ii) retaining the magnetism of the nanoparticles. Therefore, the conjugated polyelectrolytes will enable high sensitive, high-speed, high-resolution, and long-term fluorescence tracking of the magnetic nanoparticles inside biological cells. Another attractive feature of the conjugated polymers as fluorescence probes is that they are available with a range of emission colors spreading through visible to near-infrared wavelengths. Simultaneous use of the conjugated polymers with spectrally distinct emissions makes multi-channel biological fluorescence imaging feasible, and opens the way to use multiplexed optical coding to track several different MP-tagged populations of the cells at the same time. Such dual-wavelength emission properties could also serve as the basis for ratio metric sensing local chemical environment of the cells. Given the capability of surface functionalization of these nanoparticles by specific molecules (e.g., antibodies, carbohydrates, and peptide ligands), one could develop the nanoparticles that recognize the biological targets or monitor the transformation of the tumors by simultaneously noninvasive fluorescence and magnetic resonance imaging.

## Experimental section

### Materials

The magnetic nanoparticles used in this study were colloidal iron oxide prepared by co-precipitation of ferrous and ferric salts in ammonia solution^46^. The colloidal nanoparticles had negative surface charges and hydrodynamic size in the range from 30–50 nm in aqueous solution, as determined by zeta potential and dynamic light scattering experiments. Magnetization curve of the dried samples indicated that the nanoparticles were superparamagnetic, which means they could be attracted to a permanent magnet but retain no residual magnetism after the magnetic field is removed. Two conjugated polymers, i.e. fluorene-*alt*-benzene copolymer with cationic side-groups (PFN) and fluorene-*alt*-thiophene copolymer with anionic side-groups (ThPFS), were prepared by Suzuki condensation polymerization according to previous reports^47^,^48^. 2, 7-Dibromo-*N*,*N*′-diethyl-*N*,*N*,*N*′,*N*′-tetramethyl-9H-fluorene-9, 9-dipropanaminium dibromide (0.81 mmol), 1,4-phenylenebisboronic acid (0.81 mmol), and tetrakis-(triphenyl phosphine) palladium (0) (50 mg) were dissolved in a degassed mixture of 20 mL of *N*, *N*-dimethylformamide (DMF) and 40 mL of Na_2_CO_3_ solution (0.2 mol/L). The mixture was stirred at 85–90 °C for 48 h under nitrogen atmosphere. The mixture was poured into 500 ml of acetone. The precipitate was collected by centrifugation, washed by acetone three times, and then dissolved and dialyzed in distilled water using a membrane with a cutoff molecular weight of 8000–10000 g/mol for 3 days. After vacuum drying at 60 °C for 24 h, 0.32 g of the final product (PFN) was obtained. ^1^H NMR (300 MHz, D_2_O): δ 8.46–7.86 (m, 10H), 3.01 (m, 8H), 2.72 (s, 12H), 2.50–2.42 (m, 4H), 1.03 (m, 10H). ThPFS was prepared by the same procedures from an equivalent molar mixture of potassium 2,7-dibromo-9,9-bis(3′-sulfonatopropyl) fluorene (0.8 mmol) and 2,5-bis(4,4,5,5-tetramethyl-1,3,2-dioxaborolan-2-yl) thiophene (0.8 mmol) by use of tetrakis-(triphenyl phosphine) palladium (0) as the catalyst to afford 0.21 g of the final product. ^1^H NMR (300 MHz, D_2_O): 8.07–7.23 (m, 8H), 2.57 (m, 4H), 2.27–2.18 (m, 4H), 1.07 (m, 4H).

Human hepatoma carcinoma cells (Bel-7402) were provided through the courtesy of Prof. Qineng Ping (China Pharmaceutical University). Dulbecco’s modified eagle medium (DMEM), penicillin, and streptomycin were obtained from Gibco. Bovine calf serum was purchased from Sijiqing Biological Engineering Material (Hangzhou, China). 7-Aminoactinomycin D (7-AAD) was purchased from Sigma (St. Louis, MO). Trypsin and methyl thiazolyl tetrazolium (MTT) were obtained from Amresco (Solon, OH).

### Preparation of magnetic nanoparticles coated by conjugated polymer multilayers

Preparation of conjugated polymer-labeled magnetic nanoparticles was carried out at room temperature and involved three sequential steps^49^: First, the magnetic nanoparticles were suspended in aqueous solution (pH = 8.90) of tris(hydroxymethyl)aminomethane (Tris, 20 mmol/L) and NaCl (20 mmol/L) and were then added to PFN solution (1 mmol/L). The electrostatic adsorption was allowed to proceed for 30 min under ultrasonic agitation. The excess cationic conjugated polyelectrolytes, which did not electrostatically adsorb on the magnetic nanoparticles, were removed by magnetic separation/wash/re-dispersion cycles. The magnetic nanoparticles coated by monolayer of conjugated polymers were collected by permanent magnet. In the next two steps, the magnetic nanoparticles coated by bilayer and three-layer of the conjugated polymers were produced by consecutive electrostatic adsorption of ThPFS and PFN on the surface of PFN-coated magnetic nanoparticles using the same strategy to afford a final product (MP/PFN/ThPFS/PFN). The dynamic light scattering indicates that these nanoparticles were well dispersed in aqueous solutions.

*Cell Culture.* 

Bel-7402 cell line was cultured in T-25 flask at 37 °C in humidified incubator containing 5% CO_2_ atmosphere. The cell growth media is DMEM (containing inorganic salts, amino acids, vitamins, and glucose), supplemented with 10% bovine calf serum, 100 IU/mL penicillin, 100 μg/mL streptomycin, and 2 mmol/L *L*-glutamine.

### Instrumentation and measurements

*Fluorescence spectra*. Steady state fluorescence emission spectra were recorded on SLM 48000 DSCF.AB2 spectrofluorometer. The excitation wavelength was chosen to coincide with the maximum absorption of electron spectrum of the conjugated polymers.

Fluorescence quantum yields of the conjugated polymers were measured relative to quinine sulfate dihydrate in 0.05 M sulfuric acid.

#### Optical microscopy

Bel-7402 cells were seeded on cover glass in a microtiter plate with six wells (each well was used as small test tubes) at a density of 2 × 10^5^ cells per well. The cells were incubated for 48 h at 37 °C, and then washed twice with phosphate buffered saline (PBS). Then, the serum free medium (DMEM) with the fluorescence-labeled magnetic nanoparticles were added into the wells. After 4 h to 48 h incubation at 37 °C, the medium in each well was removed and the wells were washed twice with PBS. The cells were fixed with 4% of paraformaldehyde solution for 15 min, and washed three times with PBS after removal of the fixing solution. Optical and fluorescent images of the cells were captured using Leica TCS SP5 (Leica Microsystems) confocal laser scanning microscope. Bright-field optical images were collected using phase contrast or DIC (differential interference contrast) modes to enhance contrast of the samples. Fluorescence images were acquired using UV-diode laser and He/Ne laser for excitation at wavelength of 405 nm and 543 nm, respectively.

#### Flow cytometry analysis

Fluorescence intensity of the cancer cells was evaluated by excitation at 488 nm and detected through a 525/30 nm (FL1 channel) band-pass filter using a BD FACSCalibur (Becton-Dickinson Biosciences) flow cytometer. The liquid vector was Coulter Isoton III Diluent (Beckman-Coulter).

For magnetic field induced cell uptake, the 24-well plate was put on the neodymium magnets (15*5 mm column, distance between the plate and the magnets was about 3 mm). Bel-7402 cells were incubated with MPs/PFN/ThPFS/PFN for 0.5 h to 2 h, and then the wells were washed with PBS. Trypsin (0.2 mL, 0.25%) was added to each well. After trypsin digestion, the cells were suspended in 0.5 mL of DMEM, and the cell suspension was centrifuged at 2000 rpm.

#### Electron Microscopic Observation

The cancer cells and the internalized nanoparticles were investigated by using Jeol JEM-1011 transmission electron microscope (Jeol Ltd., Tokyo, Japan). The cancer cells were incubated with the fluorescence-labeled magnetic nanoparticles for 4 h, and then were prefixed with 2.5% glutaraldehyde in PBS at 4 °C overnight. After being washed with PBS three times, the cells were post-fixed with 1% osmium tetroxide dissolved in PBS for 45 min and washed by immersion in water for 10 min. After dehydration, embedding in epoxy resin, ultrathin sectioning, and staining with uranyl acetate and lead citrate, the cells were observed with TEM.

#### Cytotoxicity Assessment

Cytotoxicity of the fluorescence-labeled magnetic nanoparticles was evaluated by well-established MTT assay, which was based on the enzyme reduction activity of MTT in live cells but not in dead cells. The cell suspension in growth medium (DMEM containing 10% bovine calf serum) was seeded to a microtiter plate with 96 wells, which were arranged in a 2:3 rectangular matrix. The cells were incubated for 24 h at 37 °C, and then washed twice with PBS. Appropriate amounts of the nanoparticles were added to the growth media, and the cancer cells were incubated for 24 h or 72 h. One row of the 96-well plate (12 wells) was used as control, i.e., the cells were incubated free from the nanoparticles. 20 μL of MTT (5 mg/mL in PBS) was added to each well with 4 h post-incubation to form hydrophobic formazan in the live cells. Then the medium was removed, and 150 μL of DMSO was added to the wells to dissolve formazan. Cell viability was determined by a microplate reader (Thermo Electron Corporation).

## Results and Discussion

### Magnetic nanoparticles fluorescence-labeled by three-layer conjugated polymers

The concept of fluorescence and magnetic dual-functional nano-probe has been realized simply by alternatively electrostatic adsorption of the conjugated polymers on the surface of colloidal iron oxide nanoparticles. The as-prepared magnetic nanoparticles have negative surface charges. PFN is positively charged and blue emissive conjugated polymer with fluorescence quantum yield of 82%. Spontaneously electrostatic adsorption of PFN from Tris buffer solution (pH = 8.90) onto the colloidal iron oxide affords fluorescence-labeled magnetic nanoparticles carrying positive surface charges. The adsorption is saturated at polymer concentration of 1 mmol/L. Weakly bound PFN is removed from the surface when the dual-functional nanoparticles are rinsed and then collected by magnetic separation. For the purpose of dual-color fluorescence labeling, another fluorescence probe, i.e. ThPFS, negatively charged and green emissive conjugated polymer with a quantum yield of 62%, is electrostatically immobilized as the second polymer layer around the magnetic nanoparticles. Cationic materials, such as polymers, liposomes, oligopeptides, and inorganic nanoparticles, have been extensively used as efficient non-viral carriers for delivery genes into biological cells. In light of that, a third cationic polymer layer is envisaged and brought about by adsorption of PFN to boost the cellular uptake efficiency of the fluorescence-labeled nanoparticles. It is naturally speculated that the cellular uptake may originate from electrostatic interaction between the cationic surface charges on the nanoparticles and the negative charges on the cell membrane. The outmost layers of the final nanoparticles (MP/PFN/ThPFS/PFN) are positively charged. The coulombic repulsions give rise to stability of the colloidal nanoparticles in aqueous solution. As a result, their fluorescence under ultraviolet excitation appears homogeneously throughout the entire buffer solution. Iron element in stock aqueous solution of the nanoparticles is found at a concentration of 0.27 mg/mL by atomic absorption spectrophotometer.

The magnetic nanoparticles have zeta potential of −35 mV. After the electrostatic adsorption, the zeta potential of the nanoparticles turns to +40 mV, indicating the successful adsorption of the cationic conjugated polymer on their surface. Consequently, the zeta potential changes to −30 mV when ThPFS is electrostatically adsorbed on MP/PFN nanoparticles, confirming the assembly of secondary polymer layer. The subsequently electrostatic assembly of PFN leads to the change of zeta-potential of MP/PFN/ThPFS nanoparticles to +28 mV, verifying the effective connection between the nanoparticles and the third-layer polymer.

**Figure 2:**
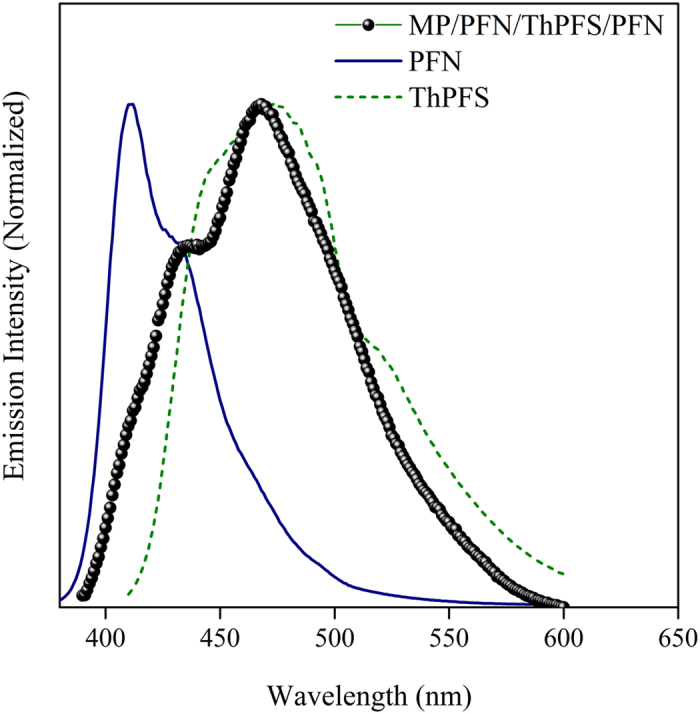
Emission spectra of the magnetic nanoparticles fluorescence-labeled by three-layer conjugated polymers (MP/PFN/ThPFS/PFN) and the corresponding conjugated polymers. The excitation wavelength is 369 nm (PFN), 400 nm (ThPFS), and 369 nm (MP/PFN/ThPFS/PFN).

PFN is high-efficiency blue-emitting material. Alternative copolymerization of fluorene with electron-deficient thiophene units is effective to reduce the optical band gap of the pi-conjugated main chains. Thus, ThPFS is green emissive with emission maximum at 473 nm, distinct from that (408 nm) of PFN. An interesting feature of such multilayer structure in the conjugated polymer-labeled nanoparticles (MP/PFN/ThPFS/PFN) is that electrostatic force brings ThPFS within close proximity of PFN. Since its fluorescence emission spectrum has good spectral overlap with electron absorption spectrum of ThPFS, PFN in its electronic excited state undergoes nonradiative decay to the ground state by Förster resonance energy transfer (FRET) to ThPFS, as a result of long-range dipole-dipole interaction between the two conjugated polymers. The fluorescence-labeled nanoparticles eventually preserve the spectral feature of ThPFS rather than that of PFN (Fig. 2).

### Cellular uptake of fluorescence-labeled magnetic nanoparticles

**Figure 3:**
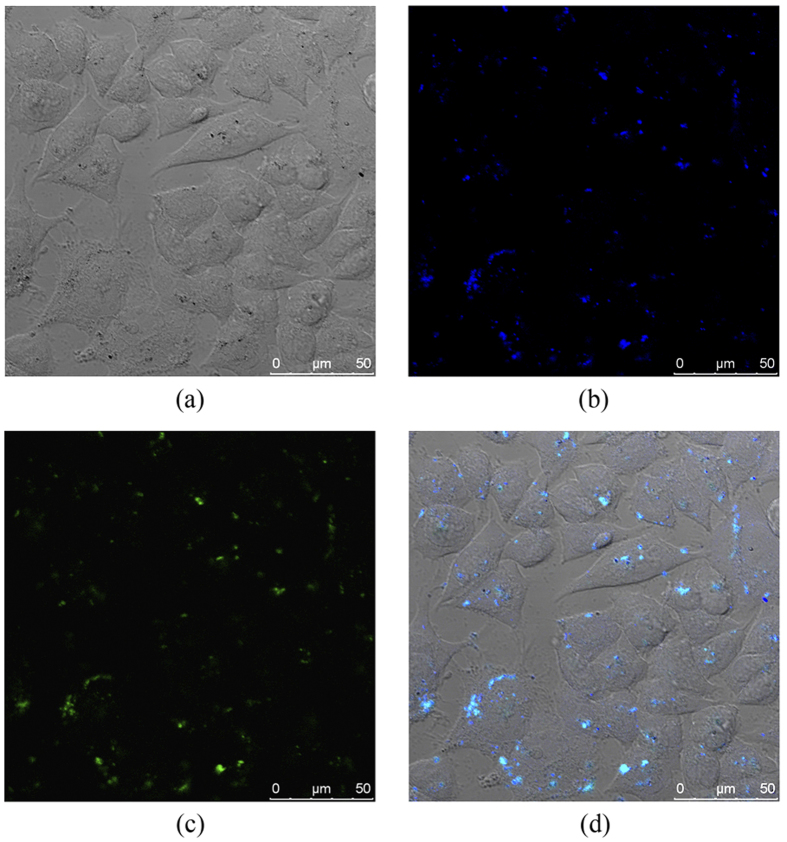
Confocal optical images of Bel-7402 cells incubated with MP/PFN/ThPFS/PFN for 4 h. (**a**) Bright-field image. (**b**,**c**) Fluorescence images at blue and green channel, respectively. (**d**) Merged image.

Advances in bionanotechnology make requests for tracking the cellular uptake of nanoparticles using non-invasive imaging methods. Human hepatoma cancer cells (Bel-7402) are incubated with 5.4 μg/mL (iron content) of the magnetic nanoparticles fluorescence-labeled by three-layer conjugated polymers (MP/PFN/ThPFS/PFN). After rinsing with abundant PBS to remove the surface-attached nanoparticles, the cancer cells are observed by confocal laser scanning microscope, and representative images are shown in Fig. 3. These cells remain viable after 4 h incubation with the fluorescence-labeled magnetic nanoparticles, as indicated by their distinct intercellular boundaries and irregular shape, suggesting strong cell-substrate adhesion. In a sense, such nanoparticles are biocompatibility. A tiny amount of large aggregates of the nanoparticles are identified as black dots or spots in bright-field image of the cells (Fig. 3a), while small aggregates or individual nanoparticles may be too small to be observed clearly.

Photoluminescence of the conjugated polymers allows direct intracellular localization of the nanoparticles by confocal optical microscopy. When the cancer cells are excited at maximum absorption wavelengths of two conjugated polymers, intense fluorescence signals at blue and green channels are observed in a majority of the cancer cells (Fig. 3b,c). It illuminates that the fluorescence-labeled nanoparticles penetrate plasma membrane of the cancer cells and translocate into the cytoplasm. Due to their large extinction coefficients and high fluorescence quantum yields, the conjugated polymers afford fluorescence brighter than cell auto-fluorescence came from endogenous structures, such as mitochondria and lysosomes. Thus, the conjugated polymers significantly enhance the sensitivity for detection and enable one to visualize extremely low concentration of the nanoparticles within the live cells. After entering into the Bel-7402 cells, the nanoparticles are unevenly dispersed in the cytoplasm. The punctuate fluorescence pattern suggests that the nanoparticles effectively internalized into the cancer cells are accumulated or aggregated within endocytic vesicular structures such as endosomes (highly dynamic membrane systems involved in transport within the cells), and/or lysosomes (membrane-bound vesicles that contain hydrolytic enzymes to break down foreign materials entering the cell).

In the merged two-dimensional image of bright-field and fluorescence pictures obtained from a single optical cross-section of Bel-7402 cells (Fig. 3d), it is notable that almost all blue and green signals exist at the same pixel location to afford cyan signals. As described earlier, resonance energy transfer can occur between PFN (the donor) and ThPFS (the acceptor) with proximal contact by electrostatic attraction during their alternative adsorptions on the surface of the magnetic nanoparticles. Since the degree of fluorescence quenching of PFN due to energy transfer is distance-dependent, extensive overlap of blue and green fluorescence signals suggests that both conjugated polymer probes tightly bind on surface of the nanoparticle during cellular internalization. Otherwise, random intracellular distribution of fluorescence signals of the conjugated polymers peeled away from the magnetic nanoparticles might be expected.

### Uptake efficiency of the fluorescence-labeled magnetic nanoparticles in cancer cells

**Figure 4:**
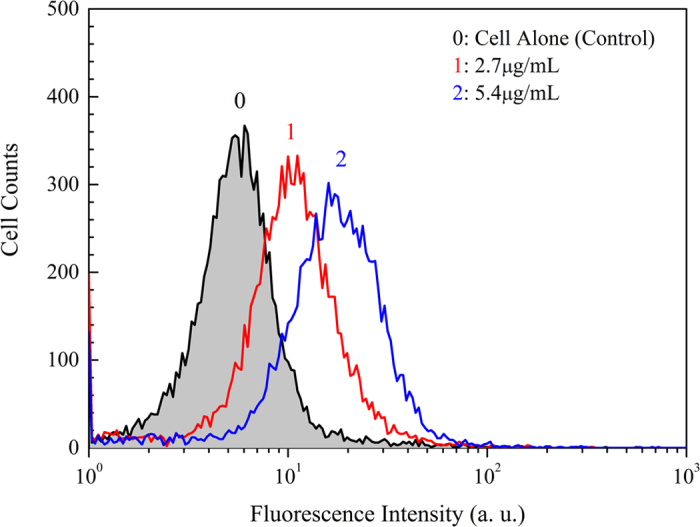
Fluorescence intensity monitored at green channel of Bel-7402 cells alone (control) and labeled by MP/PFN/ThPFS/PFN (2.7 and 5.4 μg/mL). Excitation wavelength is 488 nm.

In an attempt to quantify uptake efficiency of the fluorescence-labeled magnetic nanoparticles over a population of live Bel-7402 cells, we measure cell fluorescence using flow cytometer. The result is shown as logarithmic green fluorescence intensity on the x-axis and cell number on the y-axis (Fig. 4). The cells cultured alone, i.e. the control cells, have background fluorescence arising from endogenous fluorophores such as porphyrins, collagens, and flavins. When incubated with the fluorescence-labeled nanoparticles (2.7 and 5.4 μg/mL) for 4 h, the cancer cells display enhanced fluorescence with respect to the control. This indicates that the magnetic nanoparticles labeled by the conjugated polymers are internalized into the Bel-7402 population. Fluorescence signals are unimodal distributed. With high fluorescence quantum yields and photo-stability, the conjugated polymers can thereby serve as excellent fluorescence markers to push the detection limits of nanoparticles within live cells by using a flow cytometer, or to help observation intracellular localization of the nanoparticles by confocal microscope. It is also observed that emission intensity of the cells is nanoparticle-dosage dependent.

**Figure 5:**
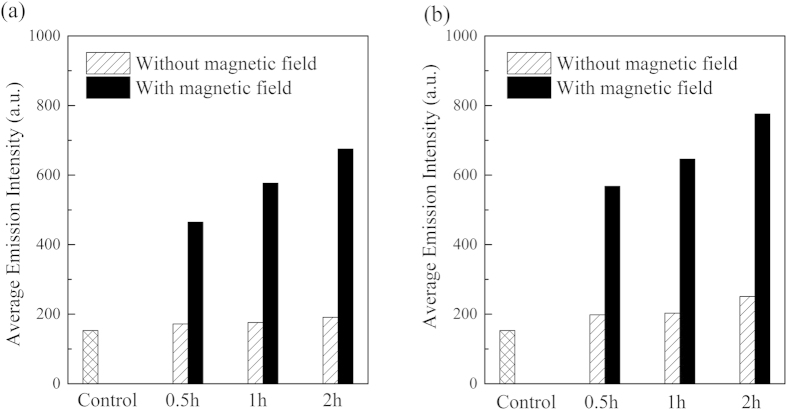
*In vitro* cellular uptake of the fluorescence-labeled magnetic nanoparticles (MP/PFN/ThPFS/PFN) in the absence and in the presence of external magnetic field. The cancer cells are incubated with the nanoparticles (**a**) 2.7 μg/mL and (**b**) 5.4 μg/mL for 0.5 h to 2 h, and then analyzed by flow cytometry. Control: Bel-7402 cells cultured in the absence of the nanoparticles.

During confocal laser scanning microscopy and flow cytometry analysis, we can observe strong fluorescence from the fluorescence-labeled nanoparticles within the Bel-7402 cells. However, the number of the nanoparticles internalized in the cancer cells are far less than that supplied during incubation. This is most likely due to the particularly resistant cell membrane. The result of flow cytometry analysis of the cancer cells with different time of incubation with fluorescence-label magnetic nanoparticles is shown in Fig. 5. Bel-7402 cells cultured in the absence of the nanoparticles (the control sample) exhibit cellular autofluorescence with an average emission intensity of 153. 0.5 h incubation with the fluorescence-labeled nanoparticles results in 12% increase of average emission intensity. 1 h to 2 h is necessary to detect expression of the fluorescence-labeled nanoparticles in the cancer cells. After 2 h incubation, fluorescence intensity of the cells increases 25%. The result indicates that the nanoparticles penetrate the cell membrane at a rather low velocity, and thereby it might be desirable to circumvent the barriers to efficient entry of current fluorescence probes into the cell and the nucleus.

The intrinsic behavior, i.e. superparamagnetism, of the magnetic nanoparticles opens up a possibility of controlling the cellular uptake by external magnetic field^50^,^51^,^52^. During incubation with the fluorescence-labeled magnetic nanoparticles (2.7 μg/mL), a magnetic field is built up as close to the cancer cells as possible. By application of an external magnetic field, high-efficient capture of the fluorescence-labeled magnetic nanoparticles by the cancer cells and retention of the nanoparticles after removal of the magnetic field is observed within 0.5 h. After 0.5 h incubation with the fluorescence-labeled magnetic nanoparticles and exposure to magnetic field, the cell fluorescence increases 204% with respect to the control sample, and the emission intensity is about 3-fold compared with that of the cells incubated in the absence of magnetic field. A 341% increase in the cell fluorescence is observed after 2 h incubation and application of magnetic field. Therefore, one is capable of “visualize”, with the help of conjugated polymer fluorescence probes, that the magnetic forces pull the magnetic nanoparticles across the plasma membrane and entering into the cytoplasm. To verify that magnetic field enhances the cellular uptake, we carry out a parallel experiment where the cancer cells are incubated with the nanoparticles with higher concentration (5.4 μg/mL), as shown in Fig. 5b. It confirms that, when exposure to magnetic field, cellular uptake of the nanoparticles significantly increases compared with that in the absence of magnetic field. Significantly promoted cellular uptake using magnetic force is attractive, because it leads to a substantially greater detectability of the cancer cells, which are illuminated by the conjugated polymers. Though magnetic force can enhance endocytosis efficiency of the nanoparticles, it may not alter the uptake mechanism but accelerate sedimentation of the nanoparticles on the cell surface, and provide extra energy for substantial internalization of the nanoparticles.

**Figure 6:**
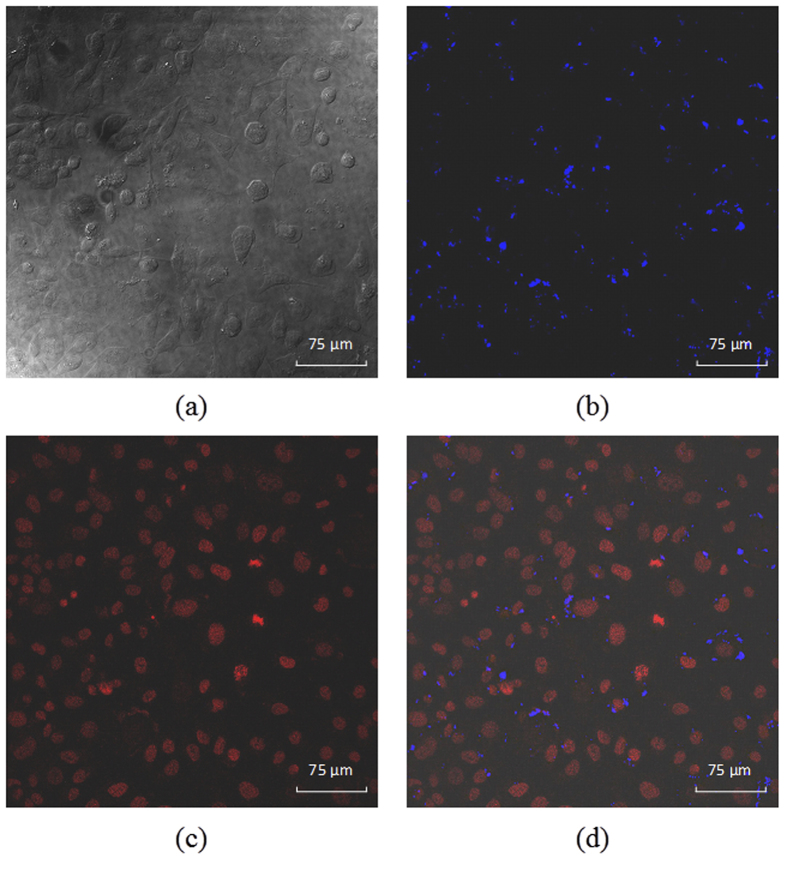
Confocal optical images of Bel-7402 cells incubated with MP/PFN/ThPFS/PFN (blue color) for 48 h. The cells are fixed with 4% of paraformaldehyde solution. The cell nuclei are stained by 7-AAD (red color). (**a**) Bright-field image. (**b**,**c**) Fluorescence images at blue and red channels, respectively. (**d**) Merged fluorescence image.

When the fluorescence-labeled magnetic nanoparticles are internalized into the cytoplasm, it is necessary to address such issues as their stability, cytotoxicity, and destination (e.g. organelle targeting). Figure 6 displays confocal optical images of the cancer cells after long-time (48 h) incubation with MP/PFN/ThPFS/PFN. Blue fluorescence is still clearly observed in the cytoplasm (Fig. 6b). It demonstrates the stability of the conjugated polymer fluorescence probes inside the cells, and it is hard to transport totally the internalized nanoparticles out of cancer cells. The cell nuclei are stained by 7-AAD, a fluorescence dye that can intercalate into double-stranded nucleic acids and emits deep red light with emission maximum at 655 nm. Therefore, its fluorescent signal can be well separated from that of the conjugated polymers (400–600 nm). 7-AAD is excluded by intact cells, and it can only penetrate through the membranes of dead cells. As a result, Bel-7402 cells are fixed with 4% of paraformaldehyde solution before nuclear staining. The nucleus exhibits high adsorption of the red fluorophores (Fig. 6c). There is no obvious evidence of the nanoparticle cytotoxicity, where the dead cells should exhibit indistinct intercellular boundaries, nuclei with undefined shape or condensed chromosomes, and blebbing. The nucleus is a desirable target of the nanoparticles, because genetic information is stored there, and meanwhile conjugated polymers have substantially be proven useful for fluorescence sensing of nucleic acids. The merged fluorescence image (Fig. 6d) from a optical cross-section of the cells shows that the conjugated polymer labeled nanoparticles (only signal in blue channel is collected for the sake of simplicity) fail to occur within nuclear regions (red in color) of the cells. Otherwise, colocalization of the red and blue signals should produce a color of magenta. A careful examination reveals that few of the nanoparticles migrate toward the nucleus periphery. Absence of nucleus targeting may due to two facts. First, escape of the nanoparticles entrapped in endocytic vesicles to the surrounding medium is difficult. Second, the nanoparticles are not small enough to cross the pores on nuclear membrane.

### Nanoparticle cellular internalization pathways

**Figure 7:**
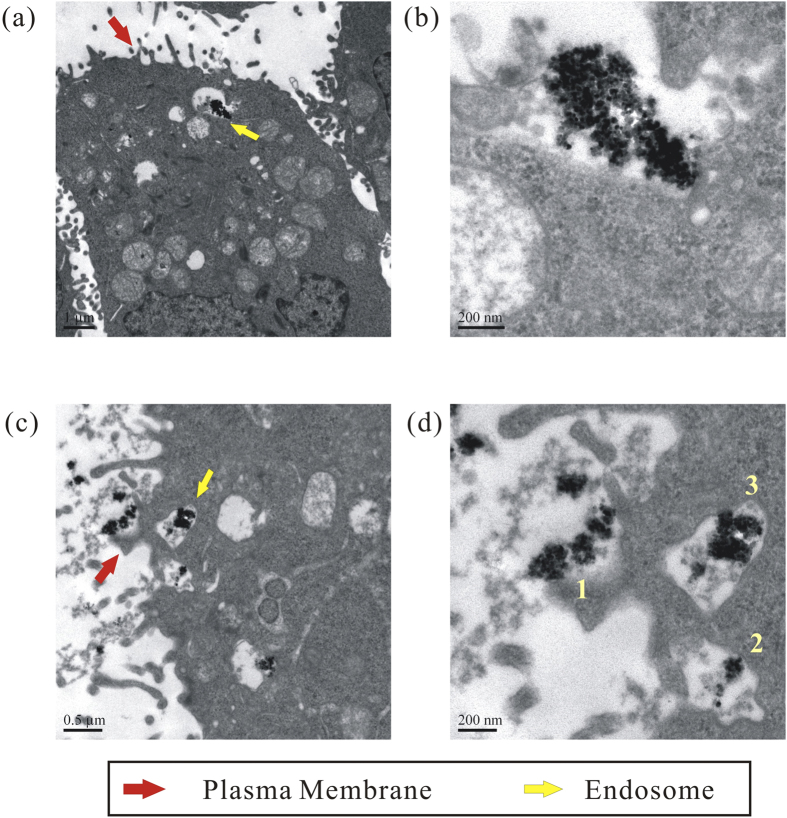
Ultrathin section electron micrographs of Bel-7402 cells after 4 h of incubation with the fluorescence-labeled magnetic nanoparticles. The nanoparticles are visible as black dots. Images (**b**) and (**d**) are magnification of the images (**a**) and (**c**), respectively. Numbers (1–3) in image d represent the nanoparticles clung to extrusion on the cell surface, encapsulated by the lipid membrane, and internalized in the vesicle.

The uptake, localization, and stability in the intracellular environment of the fluorescence-labeled magnetic nanoparticles are also examined using transmission electron microscopy. Bel-7402 cells are incubated with the nanoparticles for 4 h. Ultrathin section electron micrographs of the cells confirm intracellular accumulation of the nanoparticles. As shown in Fig. 7a, individual cells have irregular surface with protrusions and pleats, and mostly important, endosome with the loaded nanoparticles in the cytoplasm. Size of the endocytic vesicles is about 200–500 nm.

A thorough examination of the TEM images shows a number of important features. Firstly, the nanoparticles accumulated inside the endosome are observed to form aggregates (Fig. 7b). Considering the repulsion forces between positive surface charges, self-aggregation of the nanoparticles can be easily ruled out. Electrostatic attractions with oppositely charged biomolecules are expected to change surface charges of the nanoparticles, leading to intracellular aggregation. Secondly, the nanoparticles are also observed on lipid membrane of the cancer cells (Fig. 7c). The magnified image (Fig. 7d) clearly shows adhesion of the nanoparticles to cell surface, suggesting nonspecific interaction with biomolecules, for example, negatively charged phospholipids on the extrusion membrane (case 1). The same image also reveals major routes of the nanoparticles to transport from cancer cell surface to the endosome. Once the nanoparticles adhere to the cell, the lipid membrane is stimulated to form a small pocket locally around the nanoparticles/extracellular fluid (case 2), and then encloses them gradually, leading to internalization of the nanoparticles in the vesicle (case 3). The cancer cells may digest the nanoparticles through nonspecific engulfment, rather than specific receptor-mediated endocytosis.

The result also verifies that transfection efficiency of the conjugated polymer-labeled nanoparticles is limited by numerous barriers, and entrance of the nanoparticles into the cancer cells has been shown to be extremely slow. Meanwhile binding of the positively charged nanoparticles to lipid membrane permits potentially faster transfer of them into the cells in the presence of magnetic field. Delivery of the nanoparticles toward nuclear membrane and eventually entering into the nucleus should be extremely difficult without the help of external force, as confirmed by fluorescence microscopy.

In view of electrostatic or hydrophobic characteristics, the conjugated polyelectrolytes are prone to interactions with biomolecules throughout the cell internalization experiments. As conformed by confocal laser scanning microscopy and flow cytometry fluorescence analysis, the conjugated polymer coating is firmly packed on the nanoparticle surface.

### *In vitro* cytotoxicity of fluorescence-labeled magnetic nanoparticles

**Figure 8:**
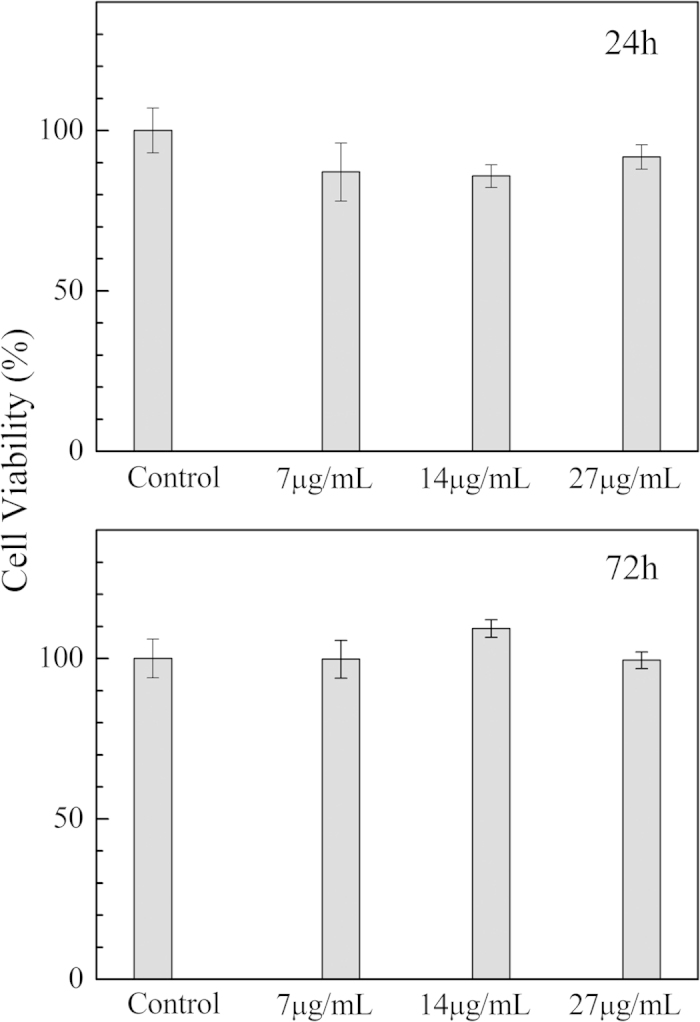
Cytotoxicity of conjugated polymer-labeled magnetic nanoparticles determined by MTT assay. Cell viability is expressed relative to the control cells.

Conjugated polymer-labeled magnetic nanoparticles can be used both as fluorescence labeling of cells and as contrast agents for magnetic resonance imaging. For any biological or clinical applications of the nanoparticles, evaluation of their toxicity to cell cultures or live animals is crucial. There has been considerable concern about nanoparticle toxicity, either coming from inherent chemical composition or arising from nanoscale effect. Numerous superparamagnetic iron oxides have already been FDA-approved for use in the clinic, as well as others undergoing clinical trials^53^,^54^. In contrast, biocompatibility of the conjugated polymers is far beyond consideration of researchers. Fig. 8 shows Bel-7402 cell viability obtained by MTT assay, which relies on mitochondrial activity of the cells and represents a parameter for the metabolic activity. At a concentration of 7 μg/mL of the fluorescence-labeled nanoparticles, cell viability decreases to a level between 80 and 90% of the control cells after 24 h incubation. For the nanoparticles with concentration up to 27 μg/mL, which is ten-fold of the dose needed for fluorescence analysis, cell viability maintains above 85% of the control. Apparently, the nanoparticles have low cytotoxicity. The result is in agreement with that obtained from microscopy where most cancer cells exhibits polygonal morphology and well-defined nuclei. When exposed to the nanoparticles for 72 h, the viabilities of cancer cells incubated with various amount of the nanoparticles are almost the same within the experimental error. Magnetic iron oxide nanoparticles taken up in the endosomes and lysosomes can be metabolized into elemental iron by hydrolytic enzymes, and then utilized by the cells^55^. Water-soluble cationic polymers have been used as non-viral carriers for the transfer of proteins, peptides, DNA, and RNA into cells and tissues. Their cytotoxicity comes from their electrostatic interaction with the cell membranes^56^, which may also be the same for the conjugated polyelectrolyte used here.

## Conclusion

Conjugated polymers with cationic and anionic side-groups can be alternatively deposited on the magnetic nanoparticles to produce fluorescent-magnetic dual functional nanoparticles with inorganic cores and three-layer fluorescent shells. The nanoparticles undergo electrostatic binding to lipid membrane, followed by non-specific uptake through endocytosis into cytoplasm. Conjugated polymers have large extinction coefficients and high fluorescence quantum yields, which enable one to visualize the nanoparticles at low concentration within live cells by fluorescence techniques, such as confocal laser scanning microscopy and flow cytometry. High efficiency delivery of the nanoparticles into cancer cells has also been realized with the aid of external magnetic field. The process of labeling live cells by such fluorescent-magnetic nanoparticles can be handled judiciously to maintain cellular viability. Another important feature of the current work is labeling of the nanoparticles by two conjugated polymers, which have different emission colors but are close to each other in space. Dual-color fluorescence labeling leads to FRET between two conjugated polymer fluorophores. FRET is appealing for tracking nanoparticle-cell interactions, which may result in notable changes in fluorescence signals during cell imaging. With the aid of magnetic field, the magnetic nanoparticles may penetrate the nuclear membrane as well. Thereby the conjugated polymers delivered into the nuclear region might be further applied as well-established DNA fluorescence probes. Combined with immune-magnetic collection using magnetic nanoparticles with affinity group specific to the antigens or acceptors on the surface of the target cells, the fluorescence-labeled magnetic nanoparticles may have potential application in the detection of circulating tumor cells in the blood.

## Additional Information

**How to cite this article**: Sun, M. *et al.* Dual-Color Fluorescence Imaging of Magnetic Nanoparticles in Live Cancer Cells Using Conjugated Polymer Probes. *Sci. Rep.* **6**, 22368; doi: 10.1038/srep22368 (2016).

## Figures and Tables

**Figure 1:**
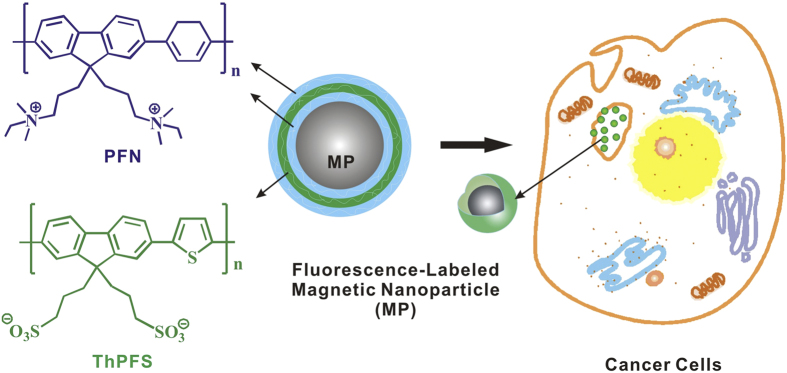
Magnetic nanoparticles (MP) are fluorescence-labeled by three layers of conjugated polymers, and then the nanoparticles are internalized by cancer cells.
